# A Meta-Analysis of Randomized Controlled Trials Concerning the Efficacy of Transversus Abdominis Plane Block for Pain Control After Laparoscopic Cholecystectomy

**DOI:** 10.3389/fsurg.2021.700318

**Published:** 2021-08-04

**Authors:** Weihua Wang, Lishan Wang, Yan Gao

**Affiliations:** ^1^Department of Thoracic Surgery, Weifang Second People's Hospital, Weifang, China; ^2^Department of Oral and Maxillofacial Surgery, Weifang Second People's Hospital, Weifang, China

**Keywords:** laparoscopic cholecystectomy, analgesia, transverse abdominis plane block, ultrasound, pain control

## Abstract

**Background and Purpose:** Transverse abdominis plane (TAP) block has been suggested to reduce post-operative pain after laparoscopic cholecystectomy (LC). However, the literature is divided on whether ultrasound (USG)-guided TAP block is effective for pain control after LC. The present meta-analysis therefore evaluated the efficacy of USG-guided TAP block vs. controls and port site infiltration for pain control after LC.

**Methods:** A comprehensive literature search of online academic databases was performed for published randomized controlled trials (RCTs) for studies published to January 31, 2021. The primary outcome analyzed was post-operative pain score at 0, 6, 12, and 24 h post-surgery, both during rest and while coughing. Secondary outcomes included morphine consumption and post-operative nausea and vomiting (PONV) incidence.

**Results:** A total of 23 studies with data on 1,450 LC patients were included in our meta-analysis. A reduction in pain intensity at certain post-operative timepoints was observed for USG-guided TAP block patients compared to control group patients. No reduction in pain intensity was observed for patients receiving USG-guided TAP block patients vs. conventional Port site infiltration.

**Conclusion:** This meta-analysis concludes that TAP block is more effective than a conventional pain control, but not significatively different from another local incisional pain control that is port site infiltration. Additional prospective randomized controlled trials are required to further validate our findings.

## Introduction

Laparoscopic cholecystectomy (LC) is currently the gold standard treatment for symptomatic gall bladder disorders, including cholelithiasis and cholecystitis (1, 2). However, LC, while minimally invasive, is associated with post-operative pain, especially within the first 24 h. This pain is routinely managed using opiates, which are associated with a number of side effects, including excessive sedation and post-operative nausea and vomiting (PONV). As these side effects may increase hospital stay durations, proper pain control and management are therefore critical for improving clinical outcomes and promoting earlier ambulation post-surgery (3–5).

Transversus abdominis plane (TAP) block is a regional anesthetic technique that has gradually become an alternative for post-operative pain control. It involves the infusion of local anesthetic into the fascial plane of the abdominal wall where the T6 to L1 nerves are found (5). Conventionally, TAP block was performed using anatomical landmarks, but ultrasound (USG)-guided TAP block has become more popular in recent years (6–10).

A previously published meta-analysis detailing seven randomized controlled trials (RCTs) showed TAP block to be effective when compared with standard analgesia in adults undergoing LC (11). However, they lacked evidence to compare the efficacy of TAP block against conventional port site infiltration for post-LC pain control. The current study aims to systematically review all available RCTs to evaluate the efficacy of USG-guided TAP block against conventional analgesia and port site infiltration in LC patients.

## Materials and Methods

### Search Strategy

This meta-analysis was performed using Preferred Reporting Items for Systematic Reviews and Meta-analysis (PRISMA) guidelines (12). A comprehensive literature search for RCTs published prior to January 31, 2021 was conducted using the following electronic databases: PubMed, Google scholar, Cochrane Library, Scopus, and TRIP. The following search terms were employed: “Transabdominal abdominis plane block” OR “Tap block” OR “Plane Block” OR “Ultrasound guided TAP block” AND “Laparoscopic cholecystectomy” AND “Pain Control” AND “Analgesic” OR “Local Anesthesia” OR “Infiltration anesthesia.” Literature cited by included studies were also manually searched for additional eligible studies. The literature search did not restrict for language.

### Study Eligibility Criteria

RCTs involving adult patients undergoing elective LC that compared the efficacy of USG-guided TAP block against either control or port-site infiltration groups were included. Studies that did not report pain outcomes were not included. Studies where full-texts were not available were not included.

### Data Collection and Analysis

All eligible studies were screened by two independent reviewers using the selection criteria listed above. Screening first entailed abstract review, followed by full-text review. Any discrepancies were settled through discussion with a third reviewer. Articles published in a language other than English were machine translated using Google Translate and considered for inclusion. The following information was extracted from each included study: number of patients, investigation groups, types of analgesia used, outcome measurements, treatments, interventions, and adverse effects.

#### Primary Outcome

The primary outcome evaluated in this study was pain control in LC patients, as measured using 1–10 rating scales such as the visual analog scale (VAS) or numerical rating scale (NRS). Measurements at 0, 6, 12, and 24 h post-operation, both at rest and while coughing, were noted.

#### Secondary Outcomes

Secondary outcomes in this study included morphine consumption and post-operative nausea and vomiting (PONV) incidence.

### Quality Assessment

Studies were assessed for quality using a modified JADAD score (13) that evaluated study methods, randomization approaches, blinding, withdrawals and dropouts, inclusion and exclusion criteria, approaches used to assess adverse effects, and statistical analysis. Scores ranged from 0 (lowest quality) to 8 (highest quality). Study quality was assessed independently by two reviewers. All discrepancies were resolved through discussion.

### Publication Bias

Publication bias was assessed using funnel plot analysis. Funnel plot asymmetry was assessed using Egger's regression test (14, 15).

### Statistical Analysis

Mean difference (MDs) with 95% confidence intervals (CIs) was calculated for the continuous outcome. Risk ratios (RR) with 95% CIs were calculated for categorical outcomes to estimate pooled findings. Study heterogeneity was evaluated using the *I*^2^ statistic. For *I*^2^ values >50%, a random-effects model was applied. For *I*^2^ values below 50%, a fixed-effect model was applied. Statistical analyses were conducted using Review Manager software (Version 5.3, Copenhagen: The Nordic Cochrane Center, The Cochrane Collaboration 2014).

## Results

### Literature Search

**Figure 1:**
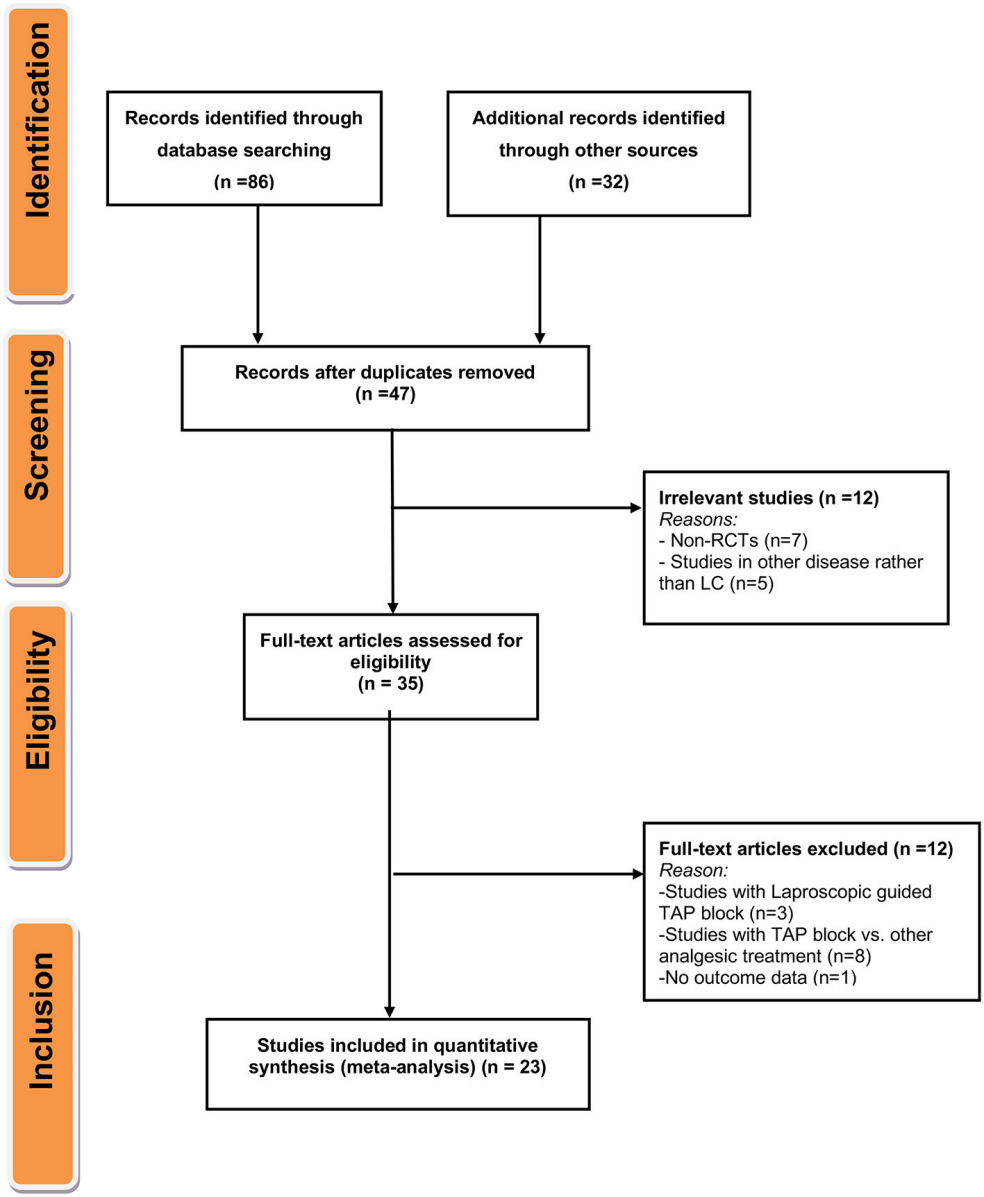
Flow diagram for the selection of studies and specific reasons for exclusion from the present meta-analysis.

Primary screening yielded 118 candidate articles. Of these, 47 underwent full-text screening and review. Ultimately, 23 studies containing data on 1,450 patients met inclusion criteria (Figure 1).

### Characteristics of Included Studies

A full summary of extracted data from included studies is presented in Table 1. Included studies were published between 2009 and 2020, with individual study samples ranging from 40 to 120 individuals. All included studies were of moderate or high quality based on JADAD Score (Table 2). Out of 23 included studies, 14 studies (16–18, 23–25, 27, 30, 31, 34–38, 40) were conducted in Caucasian individuals, with the remaining nine studies (19–22, 26, 28, 29, 32, 33) were conducted on an Asian population.

**Table 1:** Baseline and clinical characteristic of the included studies in the meta-analysis for the efficacy of transversus abdominis plane block for pain control after laparoscopic cholecystectomy.

**S. No**	**References**	**Country**	**Ethnicity**	**Groups investigated**	**TAP approach**	**Comparator**	**Treatment**	**Anesthesia**	**Post-operative analgesia**
1	Arik et al. (16)	Turkey	Caucasian	TAP Block (*n* = 24) Control (*n* = 24) Port Site Infiltration (*n* = 24)	Patients received unilateral subcostal TAP block	Patients received intravenously patient-controlled analgesia (IV PCA) or local anesthetic infiltration at port sites	0.25% bupivacaine	Propofol, fentanyl, and rocuronium	Paracetamol IV, 1 mg/kg tramadol IV, and ondansetron 0.1 mg/kg IV
2	Tulgar et al. (17)	Turkey	Caucasian	Subcostal TAP Block (*n* = 20) Control (*n* = 20)	OSTAP blocks	Control as standard analgesia plan with no block	No details provided	Propofol 2–3 mg/kg, fentanyl 100 μg, and rocuronium bromide 0.6 mg/kg. 0.6 minimum alveolar concentration sevoflurane and 0.08 μg/kg/min remifentanil infusion	1 gm paracetamol and 20 mg tenoxicam.
3	Houben et al. (18)	Belgium	Caucasian	Subcostal TAP Block (*n* = 26) Control (*n* = 26)	USG guided bilateral subcostal TAP block with 20 ml of levobupivacaine 0.375% and epinephrine 5 mg/ml_	Patients receiving 0.9% saline with epinephrine 5 mg/ml	levobupivacaine 0.375% and epinephrine 5 mg/ml	Propofol 2 mg/kg and sufentanil 0.1 mg/kg. Rocuronium 0.6mg/kg	Paracetamol and morphine in addition to the pre-operative NSAID and intra-operative ketamine and dexamethasone
4	Baral and Poudel (19)	Nepal	Asian	Subcostal TAP Block (*n* = 30) Port Site Infiltrate (*n* = 30)	Bilateral USG guided subcostal TAP block with 10 mL of 0.25% bupivacaine after the completion of surgery.	Patients receiving similar amount of local anesthetic infiltrated over all the laparoscopic port sites	0.25% bupivacaine	Fentanyl (2 mcg/kg), propofol (2 mg/kg), and vecuronium (0.8 mg/kg).	Injection Paracetamol 1 gm 6 hourly
5	Bava et al. (20)	India	Asian	TAP Block (*n* = 21) Port Site Infiltration (*n* = 21)	USG bilateral mid-axillary TAP blocks with 0.375% ropivacaine	Local anesthetic infiltration of the port site	0.375% ropivacaine	intravenously (IV)-administered propofol 2 mg/kg, fentanyl 2 μg/kg, and atracurium 0.5 mg/kg	0.5 μg/kg IV fentanyl
6	Khandelwal et al. (21)	India	Asian	TAP Block (*n* = 40) Control (*n* = 40)	USG guided STA block with 0.25% levobupivacaine both sides	Patients received 0.25% levobupivacaine through intraperitoneal route.	0.25% levobupivacaine	Propofol 2–3 mg/kg, fentanyl 2 μg/kg, and vecuronium 0.1 mg/kg	Paracetamol 15 mg/kg
7	Suseela et al. (22)	India	Asian	TAP Block (*n* = 40 Port Site Infiltration (*n* = 40)	USG guided bilateral subcostal TAP block (T) with 0.25% bupivacaine 20 ml each side	Port-site infiltration with 0.5% bupivacaine 5 ml each at 4 ports (I)	0.5% bupivacaine	Propofol 2 mg/kg IV and injection fentanyl 2 μg/kg IV.	Tramadol 1 mg/kg intravenous bolus and diclofenac 1 mg/kg intravenous infusion
8	Ortiz et al. (23)	USA	Caucasian	TAP Block (*n* = 39) Port Site Infiltration (*n* = 35)	Bilateral USG guided TAP blocks	Preincisional infiltration of the 4-trocar insertion	15 mL of ropivacaine 0.5%	fentanyl 2 Kg/kg and propofol 2.5 mg/kg.	Morphine, Fentanyl, Hydrocodone
9	Saliminia et al. (24)	Iran	Caucasian	Control (*n* = 18) TAP Block (*n* =18) Bupivacaine, sufentanil TAP Block (*n* =18) Bupivacaine	TAP block with bupivacaine and TAP block with bupivacaine plus sufentanil	TAP block with normal saline	bupivacaine 0.5% /2 mL (10 mg) sufentanil	50 mg fentanyl with lockout at 8-min	2 mg/kg intravenous (IV) propofol and 3 mg/kg IV fentanyl, tracheal intubation was facilitated by 0.6 mg/kg IV atracurium and anesthesia was maintained with 80e100 mg/kg/min propofol and 1 mg/kg IV fentanyl and 0.3 mg/kg IV atracurium administrated every 30 min.
10	Basaran et al. (25)	Turkey	Caucasian	Subcostal TAP group (*n* = 38) control group (*n* = 38).	Bilateral ultrasound-guided OSTAP blocks	Control Group	20 ml 0.25% bupivacaine	propofol 1–1.5 mg/kg and fentanyl 2 μg/kg.	tenoxicam, Morphine, Fantanyl, Tremadol
11	Ra et al. (26)	Korea	Asian	Control (*n* = 18) TAP block 0.25% (*n* = 18) TAP block 0.5% (*n* = 18	USG guided TAP block	Standard general anesthetic as control	Bilateral 15 ml of 0.25 or 0.5% L-bupivacaine after induction	Midazolam/propofol/ remifentanil	Ketorolac 30 mg and fentanyl 20 μg in the recovery room if needed, ketorolac 30 mg every 8 h on the ward
12	El-Dawlatly et al. (27)	Austria	Caucasian	TAP Block (*n* = 21) Control (*n* = 21)	USG guided bilateral TAP block	Standard general anesthetic as control	Bilateral 15 ml of 0.5 % Bupivacaine after induction	Propofol/sufentanil/ sevoflurane	PCA morphine bolus 1.5 mg IV with no basic infusion and 15 min lock out time
13	Chen et al. (28)	Malaysia	Asian	Control (*n* = 20) Subcostal TAP block (*n* = 20)	Bilateral OSTAP block using 1.5 mg/kg ropivacaine on each side	IV morphine 0.1 mg/kg	Bilateral 20 ml of 0.375% ropivacaine after induction	Propofol /fentanyl/ sevoflurane	Morphine 0.05 mg/kg, i.v., if needed
14	Shin et al. (29)	South Korea	Asian	Control (*n* = 15) TAP block (*n* = 15) Subcostal STAP block (*n* = 15)	TAP block or OSTAP block	Standard postoperative pain control alone	Bilateral 20 ml of 0.375% ropivacaine after induction	Propofol/ fentanyl/ sevoflurane	Fentanyl 25 μg, i.v. + ketorolac 30 mg in the recovery room and nalbuphine 10 mg on the ward if needed
15	Dost et al. (30)	Turkey	Caucasian	TAP block (*n* = 25) 0.25% levobupivacaine TAP block (*n* = 25) 0.5% levobupivacaine Control group (*n* = 25)	20 mL of levobupivacaine 0.5% and 30 mL 0.25% levobupivacaine was applied with a USG-guided TAP block	No TAP block or LAI was applied to the control group	levobupivacaine 0.5%/0.25%	IV propofol and 1 mcg / kg IV fentanyl	Mephridine, fentanyl, tremadol
16	Petersen et al. (31)	Denmark	Caucasian	Control (*n* = 37) TAP block (*n* = 37)	Bilateral USG guided posterior TAP blocks (20 mL 0.5% ropivacaine)	placebo blocks	Bilateral 20 ml of 0.5% ropivacaine after induction	Propofol/ remifentanil/ sufentanil	Oral acetaminophen 1 g and ibuprofen 400 mg every 6 h, morphine 2.5 mg, i.v., in the recovery room and oral ketobemidone 2.5 mg on the ward if needed
17	Venkatraman et al. (32)	India	Asian	Subcostal TAP block (*n* = 40) Control (*n* = 40)	USG guided Subcostal TAP block	Laparoscopy-guided subcostal TAP block	20 mL of 0.2%ropivacaine and 8 mg dexamethasone.	Propofol 2 mg/kg and cisatracurium 1.5 mg/kgwas	Fentanyl 2 μg/kg
18	Bhatia et al. (33)	India	Asian	Control (*n* = 20) TAP block (*n* = 20) Subcostal TAP block (*n* = 20)	Patients received an USG guided posterior TAP block using 15 mL of 0.375% ropivacaine on each side; and patients underwent a subcostal TAP block with 15 mL of 0.375% ropivacaine on each side	Patients received standard general anesthesia (control group);	Bilateral 15 ml of 0.375% ropivacaine	Propofol/morphine/ nitrous oxide/ isoflurane	Acetaminophen 1 g, i.v., every 6 h, tramadol 2 mg/kg, i.v., as an initial dose and 1 mg/kg if needed
19	Breazu et al. (34)	Romania	Caucasian	Subcostal TAP-Placebo (*n* = 25) OSTAP-Bupivacaine (*n* = 25) OSTAP-Pethidine (*n* = 25)	OSTAP-Bupivacaine (treated with 0.25% bupivacaine) and OSTAP-Pethidine (treated with 1% pethidine).	OSTAP-Placebo (treated with normal saline);	Bilateral 0.25% bupivacaine/ 20 ml of 1% pethidine	7.5 mg of midazolam, 60 min before the surgery, followed by the induction with 2 mcg/kg of fentanyl, 2 mg/kg of propofol, 0.6 mg/kg of rocuronium or 0.5 mg/kg of atracurium	1 g of IV acetaminophen at 8 h
20	Breazu et al. (35)	Romania	Caucasian	Subcostal TAP block (*n* =30) Subcoastal Placebo (*n* = 30)	Bilateral OSTAP Block receiving preoperatively with 0.25% bupivacaine.	Bilateral OSTAP Placebo receiving preoperatively with sterile normal saline	Bilateral 0.25% bupivacaine	Midazolam 7.5 mg orally 60 min before surgery, fentanyl 2 μg/kg, propofol 2 mg/kg, rocuronium 0.6 mg/kg or atracurium 0.5 mg/kg	Acetaminofen 15–20 mg/kg
21	Vrsajkov et al. (36)	Serbia	Caucasian	Subcostal TAP (*n* = 36) Control (*n* = 36)	USG guided subcostal TAP block	Control receiving standard postoperative analgesia	0.33% bupivacaine	Propofol (2.5 mg/kg), fentanyl (3 mcg/kg) and rocuronium (0.6–0.8 mg/kg)	Tramadol 1 mg/kg per 6 h
22	Tor et al. (37)	Turkey	Caucasian	TAP Block (*n* = 50) Port Site Infiltrate (*n* = 50)	USG guided TAP block with 30 ml 0.25% bupivacaine solution	20 ml 0.25% bupivacaine solution injected in three port incision sites.	Bilateral 15 ml of 0.5% bupivacaine	Propofol/fentanyl /Rocuronium	50 mg Tramadol and 800 mg Ibuprofen
23	Tolchard et al. (38)	UK	Caucasian	Subcostal STAP (*n* = 21) Port Site Infiltrate (*n* = 22)	USG guided STA block	port-site infiltration of local anesthetic	1 mg/kg bupivacaine	Propofol (2.5 mg/kg), fentanyl (3 mcg/kg), and atracurium (0.6 mg/kg),	Paracetamol(15–20 mg/kg) and diclofenac (0.5 mg/kg)

**Table 2:** Quality assessment using modified Jadad scores (Points 1–8) for the included studies in the meta-analysis.

**References**	**Was the research described as randomized?**	**Was the approach of randomization appropriate?**	**Was the research described as blinding?**	**Was the approach of blinding appropriate?**	**Was the approach used to assess adverse effects described?**	**Was there a presentation of the inclusion/exclusion criteria?^**#**^**	**Was there a presentation of withdrawals and dropouts?^**#**^**	**Was the approach of statistical analysis described?**	**Total score (max-8)**
Arik et al. (16)	1	1	0	0	0	1	1	1	5
Tulgar et al. (17)	1	1	1	1	0	1	1	1	7
Vindal et al. (39)	1	1	1	1	1	1	1	1	8
Houben et al. (18)	1	1	1	1	1	1	1	1	8
Baral and Poudel (19)	1	1	1	1	1	1	0	1	7
Bava et al. (20)	1	1	1	1	1	1	0	1	7
Khandelwal et al. (21)	1	1	1	1	1	1	0	1	7
Suseela et al. (22)	1	1	1	1	1	1	0	1	7
Ortiz et al. (23)	1	1	0	0	1	1	1	1	6
Saliminia et al. (24)	1	1	1	1	1	1	1	1	8
Basaran et al. (25)	1	1	1	1	1	1	1	1	8
Ra et al. (26)	1	1	0	0	0	1	0	1	4
El-Dawlatly et al. (27)	1	1	0	0	0	1	0	0	3
Chen et al. (28)	1	1	0	0	0	1	1	1	5
Shin et al. (29)	1	1	0	0	1	1	0	1	5
Dost et al. (30)	1	1	0	0	0	1	0	0	3
Baytar et al. (40)	1	1	0	0	0	1	1	1	5
Petersen et al. (31)	1	1	0	0	0	1	0	0	3
Venkatraman et al. (32)	1	1	1	1	1	1	0	1	7
Bhatia et al. (33)	1	1	0	0	1	1	1	1	6
Breazu et al. (34)	1	1	0	0	1	1	1	1	6
Breazu et al. (35)	1	1	0	0	1	1	1	1	6
Vrsajkov et al. (36)	1	1	1	1	1	1	0	1	7
Tor et al. (37)	1	1	1	1	1	1	1	1	8
Tolchard et al. (38)	1	1	1	1	1	1	0	1	7

# *“1” means “Yes”, “0” means “Not described”*.

For the primary outcome of pain control at rest and during coughing, out of 23 studies, 22 studies were included in the analysis for which references have been provided in Table 3. Some of the included studies had reported data only for one or two timepoints for post-operative pain score either for 0 or 6 or 12 or 24 h at rest or during coughing (17, 18, 20, 22–24, 26, 28, 30, 36–38). A single study by El-Dawlatly et al. (27) only provided the data for morphine consumption. PONV data was available for 12 studies (16, 18, 19, 21, 23, 25, 28, 29, 31, 33, 36, 37) (750 LC patients). Information on morphine consumption up to 24 h post-operation was reported by seven studies (16, 17, 23, 27–29, 31) (348 LC subjects).

**Table 3:** Summary of findings for the included studies investigating the efficacy of TAP block for pain control after LC.

**Timepoint after surgery**	**Groups**	**Status**	**No. of studies**	**Total participants**	**MD (95% CI)**	***p*-value**	***I*^**2**^ (%)**	**References**
0 h	USG TAP block vs. Control	Rest	13	850	**−1.53 [−2.21**, **−0.84]**	** <0.0001**	98	(16, 17, 21, 24, 25, 29–36)
		Coughing	9	578	**−1.72 [−2.48**, **−0.97]**	** <0.0001**	92	(16, 17, 21, 29, 31–35)
	USG TAP block vs. Port site Infiltrate	Rest	6	385	−0.26 [−1.03, 0.51]	0.51	93	(16, 19, 20, 23, 37, 38)
		Coughing	3	208	−1.28 [−2.73, 0.16]	0.08	84	(16, 20, 37)
6 h	USG TAP block vs. Control	Rest	12	730	**−1.19 [−1.62**, **−0.77]**	** <0.0001**	94	(16, 21, 24–26, 29, 31–35)
		Coughing	9	582	**−0.53 [−0.94**, **−0.13]**	**0.010**	90	(16, 17, 21, 29, 31–35)
	USG TAP block vs. Port site Infiltrate	Rest	5	322	−0.41 [−1.37, 0.56]	0.41	94	(16, 19, 20, 22, 23)
		Coughing	3	168	−0.36 [−0.97, 0.25]	0.25	0	(16, 19, 20)
12 h	USG TAP block vs. Control	Rest	8	512	**−0.75 [−1.24**, **−0.27]**	**0.002**	85	(16, 17, 21, 24, 26, 32, 33, 36)
		Coughing	6	398	−0.49 [−1.06, 0.09]	0.10	79	(16, 17, 21, 30, 33, 35)
	USG TAP block vs. Port site Infiltrate	Rest	5	322	−0.53 [−1.51, 0.45]	0.29	95	(16, 19, 20, 22, 23)
		Coughing	3	168	−1.44 [−3.25, 0.37]	0.12	93	(16, 19, 20)
24 h	USG TAP block vs. Control	Rest	12	746	**−0.53 [−0.83**, **−0.23]**	**0.0005**	86	(16, 17, 21, 24, 25, 29, 31–35)
		Coughing	9	578	**−0.37 [−0.66**, **−0.08]**	**0.01**	87	(16, 17, 21, 25, 29–31, 33, 35)
	USG TAP block vs. Port site Infiltrate	Rest	5	322	0.22 [−1.12, 1.55]	0.75	97	(16, 19, 20, 22, 23)
		Coughing	3	168	−0.36 [−0.85, 0.12]	0.14	0	(16, 19, 20)

*Bold values of mean differences (MD) represent statistically significant results (p-value < 0.05)*.

### Clinical Outcomes

#### Post-operative Pain Intensity at Rest

**Figure 2:**
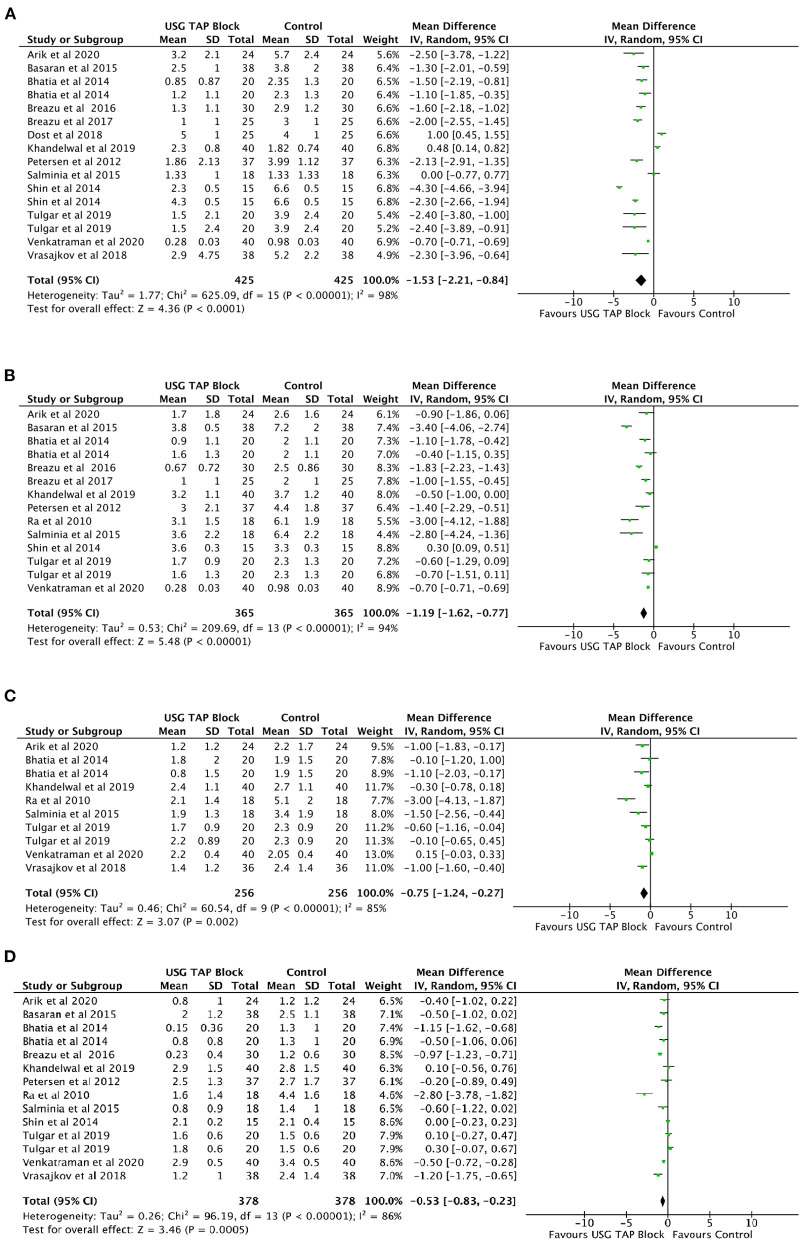
**(A–D)** Forest plot representing for Pain Intensity at Rest for USG guided TAP block vs. control group at **(A)** 0 h; **(B)** 6 h; **(C)** 12 h and **(D)** 24 h timepoints after operation.

Analysis of the included studies suggested significantly reduced pain intensity in patients receiving USG-guided TAP block relative to control group patients at 0, 6, 12, and 24 h post-operation (Figures 2A–D). However, no such reduction was noted when USG-guided TAP block patients were compared to Port site infiltration group patients (Table 3). A high degree of heterogeneity was present in all included studies at all timepoints (0 h: *I*^2^ = 98%, *p* < 0.0001; 6 h: *I*^2^ = 94%, *p* < 0.0001; 12 h: *I*^2^ = 85%, *p* < 0.0001; 24 h: *I*^2^ = 87%, *p* < 0.0001).

#### Post-operative Pain Intensity While Coughing

**Figure 3:**
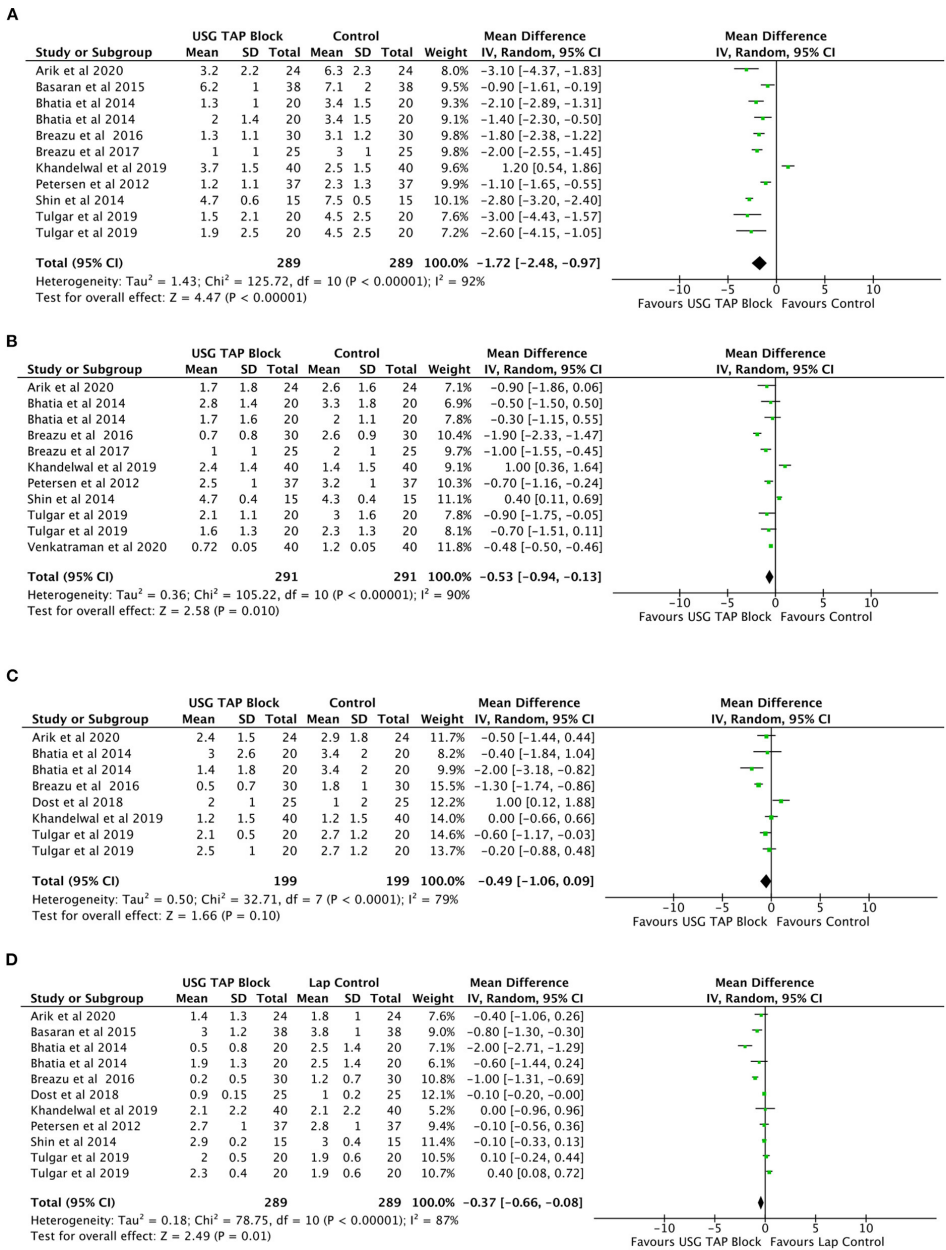
**(A–D)** Forest plot representing for Pain Intensity after operation during coughing for USG TAP block vs. control group **(A)** 0 h; **(B)** 6 h; **(C)** 12 h and **(D)** 24 h.

A significant reduction in pain intensity while coughing was observed at 0, 6, and 24 h post-operation in USG-guided TAP block patients relative to control group patients (Figures 3A–D). No significant change in pain intensity was noted at 12 h post-operation. No significant changes were noted when comparing USG-guided TAP block patients to Port site infiltration group patients at all post-operative time-point (Table 3).

#### Morphine Consumption During 24 h After Operation

**Figure 4:**
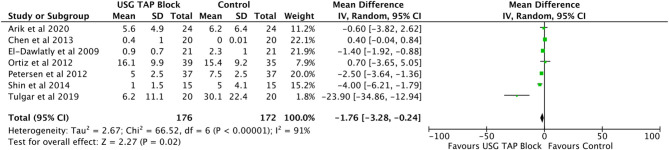
Forest plot representing for morphine consumption in the recovery room USG guided TAP block vs. control group.

Analysis of seven studies (16, 17, 23, 27–29, 31) involving 348 LC subjects showed a significant reduction in morphine consumption during the first 24 h after surgery (MD = −1.76 mg, 95% CI: −3.28 to −0.24) in patients receiving TAP blocks compared to control subjects (Figure 4). A high degree of heterogeneity was observed (*I*^2^ = 91%, *p* < 0.0001).

#### Post-operative Nausea and Vomiting (PONV) Incidence

**Figure 5:**
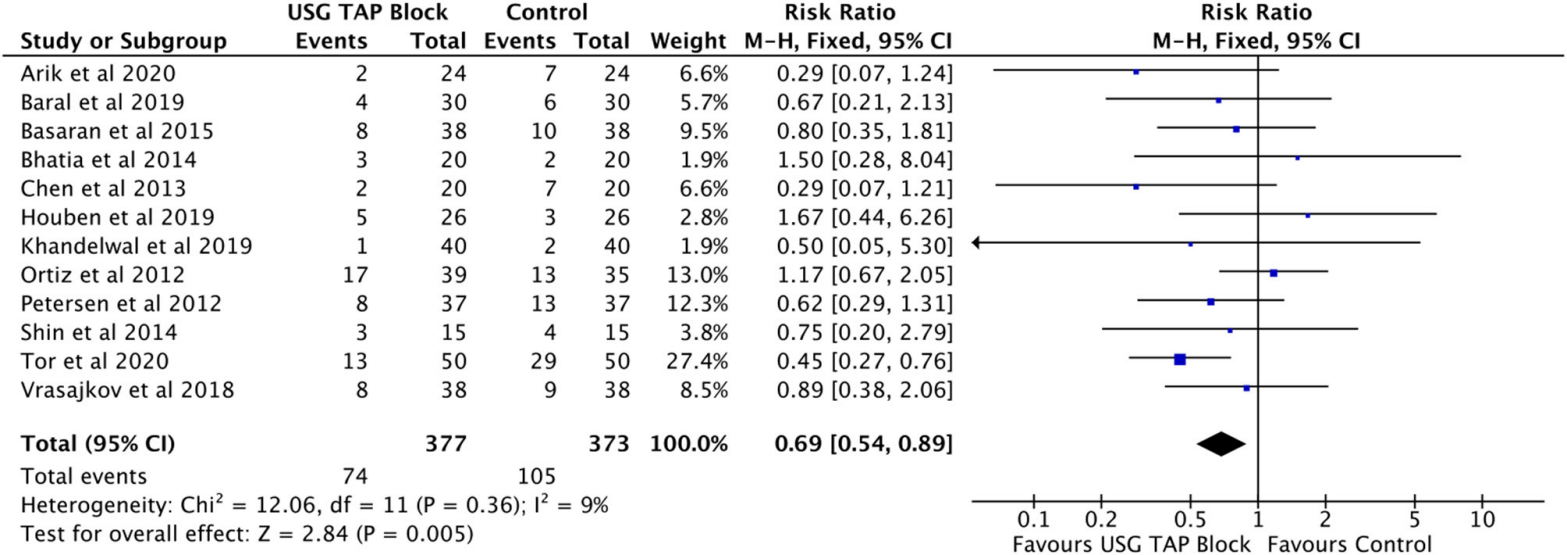
Forest plot representing for adverse events (PONV) in USG guided TAP block vs. control group.

Analysis of 12 studies (16, 18, 19, 21, 23, 25, 28, 29, 31, 33, 36, 37) involving 750 LC subjects showed a decreased incidence of PONV in patients receiving USG guided TAP blocks compared to control subjects (RR = 0.69, 95% CI: 0.54–0.89) (Figure 5). No significant heterogeneity between the studies was observed (*I*^2^ = 9%, *p* = 0.36).

### Publication Bias

No significant publication bias was detected using Funnel plots or Egger's tests for *(a) Post-Operative Pain Intensity at Rest for* all timepoints (0 h: *p* = 0.54; 6 h: *p* = 0.32; 12 h: *p* = 0.98; 24 h: *p* = 0.63); *(b) Post-Operative Pain Intensity during coughing for* all timepoints (0 h: *p* = 0.12; 6 h: *p* = 0.76; 12 h; *p* = 0.91, 24 h: *p* = 0.17)*, (c) Morphine consumption during 24 h after operation* (*p* = 0.36), *(d) Post-operative nausea and vomiting (PONV) incidence* (*p* = 0.71). All funnel plots showed symmetric shape for all the comparison.

## Discussion

This study evaluated the efficacy of USG guided TAP block for reducing pain intensity at rest and while coughing in LC patients for up to 24 h post-operation. We noted a significant reduction in pain intensity in patients who received USG-guided TAP block relative to control group patients, as well as reduced morphine consumption and incidence of PONV. However, no such reduction was noted when USG-guided TAP block patients were compared to Port site infiltration group patients at rest and during coughing at all post-operative timepoints upto 24 h.

Severe pain for LC patients generally occurs during the first 24 h post-surgery, and is thought to arise mainly from visceral tissue damage and the surgical incision (with the latter taking precedent) (41). As such, analgesic planning must focus on incisional pain rather than visceral pain. The absence of significant difference of pain control after port site infiltration advocates too for an incisional pain control. Our results showed that USG-guided TAP block led to significantly reduced post-operative pain, as well as reduced morphine consumption and PONV incidence. These findings concur with a previous analysis of seven studies by Peng et al. (11). Recent RCTs have suggested that USG-guided TAP block plays an important role in multimodal pain therapy through proper visualization and improved accuracy. However, few studies have compared the effects of subcoastal TAP block with those of posterior TAP block. As such, our meta-analysis included RCTs that looked at both USG-guided TAP blocks and oblique subcostal TAP blocks. Nonetheless, we noted high heterogeneity between studies, suggesting that our meta-analysis results need to be treated with caution.

Our study has several limitations: (1) included studies had relatively small sample sizes; (2) extended follow-up data on chronic pain, long-term analgesic use, and adverse events was lacking; (3) multiple pain score scales were used; (4) a diverse range of anesthesia dosages were administered across the studies, and (5) insufficient study numbers for important factors such as laproscopic guided TAP block and fentanyl, tramadol, and opioid consumption, thereby precluding subgroup analysis.

## Conclusion

This meta-analysis concludes that TAP block is more effective than a conventional pain control, but not significatively different from another local incisional pain control that is port site infiltration. Additional prospective randomized controlled trials are required to further validate our findings.

## Data Availability Statement

The raw data supporting the conclusions of this article will be made available by the authors, without undue reservation.

## Author Contributions

WW conceived and designed the study. WW and LW did literature search and analyzed the data. YG wrote the paper, reviewed, and edited the manuscript. All authors have read and approved the final manuscript.

## Conflict of Interest

The authors declare that the research was conducted in the absence of any commercial or financial relationships that could be construed as a potential conflict of interest.

## Publisher's Note

All claims expressed in this article are solely those of the authors and do not necessarily represent those of their affiliated organizations, or those of the publisher, the editors and the reviewers. Any product that may be evaluated in this article, or claim that may be made by its manufacturer, is not guaranteed or endorsed by the publisher.
